# No association between history of psychiatric treatment and postoperative weight reduction after bariatric surgery

**DOI:** 10.1007/s40519-024-01645-9

**Published:** 2024-03-15

**Authors:** Magdalena Kozela, Urszula Stepaniak, Karolina Koziara, Izabela Karpińska, Piotr Major, Maciej Matyja

**Affiliations:** 1 Department of Epidemiology and Population Studies, Institute of Public Health, Jagiellonian University Medical College, 8 Skawinska St., 31-066 Krakow, Poland; 2 2nd Department of General Surgery, Jagiellonian University Medical College, Krakow, Poland

**Keywords:** Bariatric surgery, Obesity management, Psychiatric aspects, Weight loss

## Abstract

**Purpose:**

The objective of the study was to assess whether the history of psychiatric treatment was associated with (1) body weight and BMI on admission for bariatric surgery, (2) weight loss > 5 kg prior to bariatric surgery, and (3) postoperative body weight reduction.

**Methods:**

Data from medical records of all consecutive patients admitted for surgical treatment of obesity in the 2nd Department of General Surgery Jagiellonian University Medical College were obtained. There were 1452 records of patients who underwent bariatric surgery between 2009 and 2021 included in the study.

**Results:**

History of psychiatric treatment was found in 177 (12%) of the sample and was inversely associated with body weight and BMI on admission for surgery in women. Men with history of psychiatric treatment were 54% less likely to lose > 5 kg before the surgery (OR = 0.46 95% CI = 0.24–0.88). Both in men and women %TWL did not differ significantly by history of psychiatric treatment (Me: 40.7 vs. 45.9; *p* = 0.130 and Me: 27.0 vs. 23.9; *p* = 0.383, respectively). After adjustment for covariates no association was found between history of psychiatric treatment and body weight reduction one year after surgery.

**Conclusion:**

Although men with preoperative history of psychiatric treatment had lower odds of losing weight before the surgery, psychiatric treatment did not differentiate the effectiveness of bariatric treatment in 1 year of observation. Bariatric surgery appears to be an effective obesity care for people treated for mental disorders.

*Level of evidence:* III *Evidence obtained from cohort or case-control analytic studies.*

## Introduction

There is an international consensus in guidelines for bariatric surgery that psychological assessment and care for patients with obesity includes all: pre-operative, early peri-operative, and post-operative periods [1–4]. The relationships between bariatric treatment and mental disorders are complex and multidimensional. There are studies indicating that bariatric surgery contributes to reducing symptoms of psychiatric disorders in 3 years after surgery [5]. This was in line with the long-term improvement observed in the severity of anxiety and depressive symptoms in bariatric patients [6, 7]. However, increased rates of de novo diagnoses of depression following surgery [7] as well as the evidence of no effect of bariatric surgery on mood disorders were also reported [8]. There is a high comorbidity of obesity with bipolar disorder and according to current evidence, bariatric surgery is considered appropriate and may be beneficial for carefully selected patients with bipolar disorder [9, 10]. It was also found that bariatric patients show higher suicide rates than the general population [11, 12].

Patients suffering from psychiatric disorders require intensified monitoring to assess their eligibility for the bariatric treatment [4]. According to the meta-analysis of 59 studies reporting the prevalence of preoperative mental health conditions, the most common mental health problem in patients seeking and undergoing bariatric treatment was depression (19%; 95% CI = 14–25%) and binge eating disorder (17%; 95% CI = 13–21%) [13]. Potential contraindication to bariatric surgery does not result from mental disorders themselves, but it may occur in patients who are not able to sufficiently control the disorder despite treatment and pharmacotherapy [4, 14]. Thus, the presurgical psychological assessment is not only focused on identifying psychiatric illness, but also on evaluation of the mental health stability and the ability to cope-up with the postsurgical physical, psychosocial, and lifestyle stressors [4, 14, 15]. Patients with psychiatric disorders were found to have impaired adherence to treatment before bariatric procedure [16]. Lack of cooperation with the patient may be a relative contraindication to the surgery [1, 3]. It is postulated to support careful screening and clarification of psychiatric medications to ensure the effectiveness and safety of the treatment, especially in patients without a formal psychiatric diagnosis [14]. The effectiveness of bariatric treatment is different in various groups and one of the determinants is the presence of mental disorders [5]. It may negatively affect eating behavior after bariatric surgery and patients’ adherence and as a consequence reduce the effectiveness of bariatric treatment [17, 18]. Muller et al. found that mental illness has a negative impact on weight loss in bariatric patients in a 4-year follow-up [19]. Worse effect of body weight reduction by almost 4% TWL in patients with mental disorders was observed also in other study of nearly 2500 participants. However, the effect, although smaller in patients with mental disorders, does is not necessarily have to be insufficient. Although improvement in body weight and quality of life were smaller, the effect in this group was still beneficial [17]. In other studies between 22 and 32% patients were taking psychiatric medications before surgery but psychiatric medication use was not associated with poorer effect 1 year after bariatric surgery [20, 21]. However, a meta-analysis of 27 studies reporting associations between preoperative mental health conditions and postoperative outcomes indicated inconclusive evidence regarding the association between preoperative mental health and postoperative weight loss [13].

Since the results on the relationship between mental problems and the effectiveness of bariatric procedures are ambiguous; the proportion of people with obesity [22, 23] and the frequency of mental disorders are parallelly increasing in general population, more research that add new knowledge on these relationship is necessary.

The objective of the present study was to assess whether the history of psychiatric treatment was associated with (1) body weight and BMI on admission for bariatric surgery, (2) weight loss > 5 kg prior to bariatric surgery, and (3) postoperative body weight reduction.

## Materials and methods

### Studied group

Data from medical records of all patients admitted for surgical treatment of obesity in the 2nd Department of General Surgery Jagiellonian University Medical College, were obtained. It is one of the biggest in the country and the main center in the region of Lesser Poland which specializes in obesity surgery with nearly 2000 patients operated on so far, and approximately 1000 outpatient consultations carried out annually. There were 1452 records of patients who underwent bariatric surgery between 2009 and 2021 included in the study. The analysis involved secondary analysis of pseudonymized data.

### Data

All medical records of patients who underwent bariatric surgery between 2009 and 2021 were reviewed and data on (1) body weight in kilograms and body height in centimeters on admission for surgery; (2) maximum body weight (declared by the patient maximum body weight); (3) body weight one year after surgery were obtained. The body mass index on admission was calculated as one’s weight in kilograms divided by the square of height in meters. The weight loss in preoperative period was calculated as a difference between the maximum body weight and body weight on admission for surgical procedure. The categorical variable indicating patients whose body weight decreased by 5 kg or more prior to the surgery, was derived. Postoperative body weight reduction was defined as the difference between maximum body weight and body weight 1 year after surgery. Percentage of total weight loss (%TWL) was calculated as ((maximum body weight minus body weight 1 year after surgery)/maximum body weight) × 100.

Information on the history of psychiatric treatment was recorded. Qualification for the 'history of psychiatric treatment' category referred to the period of approximately half a year before bariatric surgery and included conditions that were patients’ current health problem (recorded either during the qualification for the bariatric surgery or during 6-month preparatory period, during which additional consultations, including psychological consultations were done). Three main categories of psychiatric disorders based on medical records were recognized: (1) depression, (2) major mental disorders i.e.: schizophrenia or condition after suicide attempt or severe anxiety or bipolar disorder, (3) minor mental disorders, i.e.: low mood or low self-esteem, or in psychotherapy for other reason.

Further, information on covariates such as age, sex, information about current cigarette smoking (yes/no), and the duration of the obesity was obtained.

### Statistical analysis

Continuous variables were presented as mean (standard deviation) or median (Q1–Q3) as appropriate. The Shapiro–Wilk test was used to examine the normality of the distribution of each variable. Categorical variables were reported as number (percentage). Distributions of continuous variables by history of psychiatric treatment were compared using t-test for normally distributed variables or using *U* Mann–Whitney test for other distributions. Associations between categorical variables were assessed using Chi^2^ test. The multivariable logistic regression was used to assess the relationship between history of psychiatric treatment and (1) body weight and (2) body weight loss, with adjustment for covariates. The analysis was performed with stratification by sex as the differences in the distribution of other variables by psychiatric treatment were observed in men and women. Analysis was performed using Statistica v.13.1 StatSoft, Polska, Kraków, Stata Statistical Software: Release 14.2 College Station, TX: StataCorp LP. P-values < 0.05 were accepted as statistically significant.

## Results

There were 952 records of women and 500 records of men who underwent bariatric surgery between 2009 and 2021 included in the analysis. Mean age of participants was 43.2 years (SD = 10.58 years) (Table 1). Median time of obesity duration was 20 years. Mean body weight and BMI on admission were 131.5 kg (SD = 23.15) and 47.6 kg/m^2^ (SD = 6.91), respectively. Weight loss before the surgery was observed in 1070 participants (73.7%), and median weight loss in the studied group was 5 kg. There were 177 (12.2%) patients with history of psychiatric treatment. Among them depression was diagnosed in 104 (58.7%) patients, in further 33 (18.7%) patients one of major mental disorders was diagnosed; there were 23 (13%) patients with diagnosis of both depression and one of major mental disorders and there were 17 (9.6%) patients with minor mental disorder (Table 1).

**Table 1:** Characteristics of the studied sample, *N* = 1452

Characteristic		
Age [years] (*x, sd*)	43.2	10.58
Sex (men; *n*,%)	500	34.4
Body weight on admission for surgery (*x, sd*) [kg]	131.5	23.15
BMI on admission for surgery (*x, sd*) [kg/m^2^]	47.6	6.91
Weight loss before surgery (*Me*, Q1–Q3) [kg]	5	0–10
Weight loss > 5 kg before surgery (*n*, %)	658	45.4
Body weight one year after surgery (*x, sd*) [kg] (*n* = 601)	94.8	19.36
Postoperative body weight reduction (*x, sd*) [kg] (*n* = 601)	44.2	31.0
TWL (*Me*, Q1–Q3) [%] (*n* = 601)	31,9	19.4–43.5
Duration of obesity (*Me*, Q1–Q3) [years]	20	10–29
Current smoking (*n*, %)	213	14.7
History of any psychiatric treatment (*n*, %)	177	12.2
Including:		
Depression	104	58.7
Depression and one of major mental disorder	23	13.0
Major mental disorder	33	18.7
Minor mental disorder	17	9.6

*x* average, *sd* standard deviation, *n* number of participants. *Me* median, Q1–Q3—interquartile range

In Table 2, distribution of studied characteristics by history of psychiatric treatment in men and women was presented. Compared to women with no history of psychiatric treatment, women with history of psychiatric treatment were slightly older (45.2 vs.42.0 years *p* = 0.001), had lower mean body weight (117 kg vs. 122 kg; *p* = 0.005) and BMI (42.9 kg/m^2^ vs. 44.1 kg/m^2^; *p* = 0.026) on admission for surgery. In men, weight loss > 5 kg before surgery was less frequent in patients with history of psychiatric treatment (33.3% vs. 51.4%; *p* = 0.021).

**Table 2:** Distribution of patients’ characteristics by history of psychiatric treatment and by sex

Women	History of psychiatric treatment				
	Yes, *n* = 132		No, *n* = 820		*p*
Age [years] (x,sd)	45.2	10.18	42.0	10.62	0.0014
Body weight on admission for surgery (Me, Q1–Q3) [kg]	117.0	107.75–127.5	122.0	111.0–132.0	0.005
BMI on admission for surgery (x,sd) [kg/m^2^]	43.6	5.50	44.8	6.08	0.028
Weight loss before surgery (Me, Q1–Q3) [kg]	5.0	0–11	4.0	0–9.5	0.454
Weight loss > 5 kg before surgery (*n*, %)	61	46.2%	348	42.5%	0.423
Body weight one year after surgery (x,sd) [kg] (*n* = 403)	92.8	17.64	94.7	18.68	0.481
Postoperative body weight reduction (x,sd) [kg] (*n* = 403)	− 31.51	23.17	-36.01	27.67	0.269
%TWL (Me, Q1–Q3) (*n* = 403)	23.9	12.7–38.0	27.0	16.8–39.7	0.383
Duration of obesity (Me, Q1–Q3) [years]	21	11–30	18	10–28	0.473
Current smoking (n,%)	24	18.2%	105	12.8%	0.094
Men	Yes, *n* = 45		No, *n* = 455		*p*
Age [years] (x, sd)	45.2	12.11	44.4	10.26	0.611
Body weight on admission for surgery (Me, Q1–Q3) [kg]	140.0	132–153	146.0	133–164	0.082
BMI on admission for surgery (x,sd) [kg/m^2^]	45.6	6.46	46.7	6.71	0.297
Weight loss before surgery (Me, Q1–Q3) [kg]	5.0	0–8	6.0	0.5–12.2	0.106
Weight loss > 5 kg before surgery (n,%)	15	33.3%	234	51.4%	0.021
Body weight one year after surgery (x,sd) [kg] (*n* = 198)	101.2	22.08	94.9	20.84	0.247
Postoperative body weight reduction (x,sd) [kg] (*n* = 198)	− 51.2	26.96	− 63.0	31.06	0.142
%TWL (Me, Q1–Q3) (n = 198)	45.9	28.0–45.9	40.7	29.0–50.3	0.130
Duration of obesity (Me, Q1–Q3) [years]	18.5	14–35	20.0	10–29	0.504
Current smoking (n,%)	12	26.7%	72	15.9%	0.065

*x* average, *sd* standard deviation, *n* number of participants. *Me* median, Q1–Q3—interquartile range, *p*-values < 0.05 were accepted as statistically significant

**Fig. 1:**
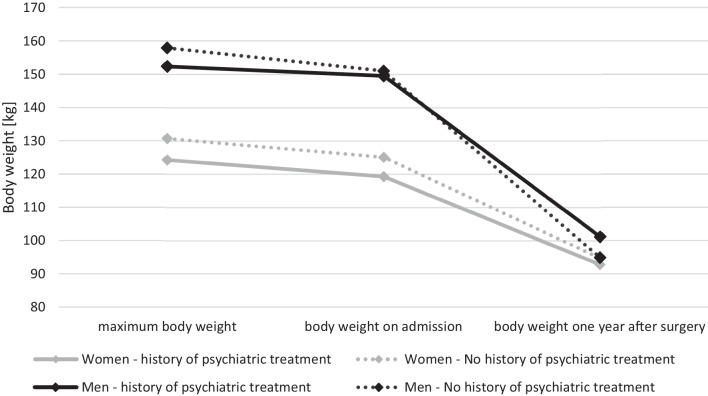
Mean maximum body weight, body weight on admission for surgery and body weight 1 year after bariatric surgery by history of psychiatric treatment and sex (all complete records)

Although the maximum body weight and body weight on admission were slightly higher in patients with no history of psychiatric treatment, the mean body weight one year after surgery was very similar in both groups in men and women (shown in Fig. 1).

In women, after adjustment for age and current smoking, history of psychiatric treatment was inversely associated with body weight on admission for surgery (Table 3). Women with a history of psychiatric treatment underwent surgery with a body weight lower by nearly 4 kg and with a BMI lower by 1.3 kg/m^2^. No association was found between history of psychiatric treatment and weight loss > 5 kg before surgery as well as postoperative body weight reduction and %TWL.

**Table 3:** Association between the history of psychiatric treatment and body weight, BMI on admission for surgery, postoperative body weight reduction, %TWL, and weight loss > 5 kg before surgery in women, *n* = 952

	Model	B	SE	*R*^2^	*p*
Body weight on admission for surgery	a	− 3.79	1.65	0.01	0.022
BMI on admission for surgery	a	− 1.30	0.57	0.01	0.022
Postoperative body weight reduction	a	− 3.52	4.08	0.01	0.389
	b	1.88	3.07	0.45	0.541
%TWL	a	− 0.98	2.59	0.01	0.704
	b	2.05	2.26	0.30	0.365

		OR	95% CI		*P*
Weight loss > 5 kg before surgery	a	1.20	0.83	1.74	0.341
	b	1.13	0.77	1.64	0.540

Model a: adjusted for age, and current smoking

Model b: adjusted for age, and current smoking and body weight before the surgery

After adjustment for age, current cigarette smoking and initial body weight, history of psychiatric treatment was not related to body weight or BMI on admission for surgery, postoperative body weight reduction and %TWL (Table 4). However, men with history of psychiatric treatment were over 50% less likely to lose > 5 kg before the surgery. The association was independent of age, current smoking, and body weight before the surgery.

**Table 4:** Association between the history of psychiatric treatment and body, BMI on admission for surgery, postoperative body weight reduction, %TWL and weight loss > 5 kg before surgery in men; *n* = 500

	Model	B	SE	*R*^2^	*p*
Body weight on admission for surgery	a	− 4.69	3.46	0.05	0.176
BMI on admission for surgery	a	− 1.12	1.05	0.001	0.285
Postoperative body weight reduction	a	− 12.46	7.94	0.03	0.118
	b	− 10.72	5.77	0.49	0.064
%TWL	a	− 6.09	4.00	0.03	0.130
	b	− 5.43	3.71	0.26	0.145
		OR	95% CI		p
Weight loss > 5 kg before surgery	a	0.47	0.25	0.90	0.023
	b	0.46	0.24	0.88	0.018

Model a: adjusted for age and current smoking

Model b: adjusted for age and current smoking and body weight before the surgery

## Discussion

Our study indicates that history of psychiatric treatment was related to lower body weight and lower BMI in women undergoing bariatric surgery. In men, body weight on admission for surgery did not significantly depend on psychiatric treatment; however, the association was manifested by lower odds of weight loss > 5 kg before the procedure. Psychiatric treatment was not related to the postoperative body weight reduction. The results suggest that bariatric treatment may be an effective method of obesity management, regardless of the mental disorders treatment. The results are in line with some other studies. In a prospective cohort study of 341 Canadian patients, diagnosis of psychiatric disorder was not associated with weight loss one year after surgery [24]. Effectiveness of surgery measured by weight loss was similar in patients with and without mental health problems. Similarly, in the study of 199 patients who were observed for 2–3 years after bariatric surgery, pre-surgery psychiatric disorders were not related to weight change [5]. On the other hand, in study by Vermeer et al. in bariatric patients, in whom 163 had preoperative diagnosis of psychiatric disease and 2362 had no such diagnosis, total weight loss 1 to 4 years after surgery was significantly lower in the psychiatric group [17]. However, the difference was rather small—about 5% lower weight loss in patients with diagnosis of mental disorders. The conclusion of this study was that the effect of surgery was sufficient in the psychiatric group as the average total weight loss was 20.9% after 4 years. In another study of 11,159 patients from Ontario Bariatric Registry, preoperative psychiatric diagnosis was associated with lower weight loss in 1 year after surgery compared to patients without psychiatric diagnosis, but this association was explained by baseline psychological distress [25]. In a qualitative systematic review, poor mental health appeared to be a barrier for successful weight loss among patients with obesity however, the findings referred only to one method of bariatric procedure (intragastric balloon) [18]. The inconsistency in findings on relation between preoperative psychiatric diagnosis and effectiveness of bariatric treatment is probably related to different methods used (e.g., different size of sample studied, different methods of evaluation of mental problems or assessment of weight loss outcome).

The lack of the association between psychiatric treatment and weight loss one year after surgery in our study, although consistent with other findings, to some extent may depend on the fact that data on body weight one year after surgery were not recorded for all patients (41% of the studied sample). Thus, conclusions about the relationship between the effectiveness of the bariatric treatment and the history of psychiatric treatment may be biased. However, the proportion of follow-up data on body weight is similar to this obtained by other authors [25]. Therefore, with some caution, it can be concluded that psychiatric treatment is not related to the 1-year effectiveness of the bariatric procedure.

We observed the association between psychiatric treatment and the lower odds of weight loss before the surgery in men. On the one hand, it may be the consequence of psychiatric disorders, which results in lower motivation and worse adherence to presurgical lifestyle modifications [16]. But on the other hand, an unfavorable effect of psychiatric medication use on body weight is well established [26–29]. In women, the association between history of psychiatric treatment and reduced odds of pre-surgery weight loss was not confirmed. However, despite obesity qualifying for bariatric surgery, women with history of psychiatric treatment had on average lower body weight on admission for surgery, than those with no psychiatric treatment. This observation is inconsistent with the results of studies that indicate body weight gain as an adverse effect of mental disorders treatment [26–29]. However, not confirming such a relationship may result from the fact that the prevalence of preoperative psychiatric treatment in our sample was rather low. Other studies indicated up to 5 times higher frequency of preoperative psychiatric treatment [4, 25, 30]. Also, Polish preliminary data on the prevalence of mental disorders in patients qualified for the nationwide multi-center pilot of bariatric obesity treatment program (KOS-BAR) showed that 12.1% patients were in psychiatric treatment but there were additional 11.7% with de novo diagnosis [31].

### Strengths and limits

In addition to the limitation related to incomplete body weight data one year after surgery, there are some other limitations that should be considered. Data were collected for clinical purposes and the measurement standardization process was not as strict as in the case of examining patients for scientific purposes only. However, data come from one department where the same agreed procedures were applied. Further, the analysis could only be performed on the data collected routinely in medical records. For this reason, much information, including data on psychiatric treatment, is not very detailed and does not differentiate psychiatric diagnoses and times when it was done. Further, the information on mental health 1 year after surgery was unavailable so the potential improvement in mental health was impossible to assess. It cannot be excluded that some observed differences between men and women depend on the indications for which psychiatric treatment was used. However, there are strengths of the analysis that should be emphasized. A large group of all consecutive patients who underwent surgical treatment of obesity for over 12 years in the biggest center performing bariatric procedures in Lesser Poland. The associations were assessed with adjustment for potential confounders.

## Conclusion

Despite the fact that men with preoperative history of psychiatric treatment had lower odds of losing weight before the surgery, psychiatric treatment did not differentiate the effectiveness of bariatric treatment in 1 year of observation. Adherence to the guidelines for a detailed mental health assessment before surgery is crucial, but bariatric surgery also appears to be an effective obesity care for people treated for mental disorders.

## Data Availability

The raw data supporting the conclusions of this article may be made available by the authors, upon reasonable request.
